# Analyzing electronic medical records to extract prepregnancy morbidities and pregnancy complications: Toward a learning health system

**DOI:** 10.1002/lrh2.10473

**Published:** 2024-11-26

**Authors:** Yitayeh Belsti, Lisa Moran, Aya Mousa, Rebecca Goldstein, Daniel Lorber Rolnik, Mahnaz Bahri Khomami, Mihiretu M. Kebede, Helena Teede, Joanne Enticott

**Affiliations:** ^1^ Monash Centre for Health Research and Implementation (MCHRI), Faculty of Medicine, Nursing and Health Sciences Monash University Melbourne Victoria Australia; ^2^ Monash Health Melbourne Victoria Australia; ^3^ Department of Obstetrics and Gynaecology, School of Clinical Sciences Monash University Melbourne Victoria Australia; ^4^ German Cancer Research Center (DKFZ) Heidelberg Germany

**Keywords:** comorbidities, gestational diabetes mellitus, learning health system, natural language processing

## Abstract

**Introduction:**

Preexisting and pregnancy‐related medical conditions frequently co‐occur, leading to multimorbidity (≥2 morbidities) in pregnant women, and much of this information is in semi‐structured format in electronic medical records (EMRs). The aim was to advance the learning health system as a platform for automating information extraction from EMRs and to uncover the prevalence of common morbidities during pregnancy and their association with pregnancy‐related complications.

**Methods:**

This study included 48 502 pregnant women attending Monash Health maternity hospitals from 2016 to 2021. Natural language processing (NLP) was used to extract morbidities from semi‐structured text in EMRs. Chi‐squared tests were used to assess the association between morbidities of gestational diabetes mellitus (GDM) and other pregnancy complications. The *k*‐means clustering algorithm identified clusters of comorbid conditions associated with GDM.

**Results:**

The most common comorbidities during pregnancy were vitamin deficiency (14 019; 28.9%), overweight (13 918; 28.7%), obesity (11 026; 22.7%), anemia and other blood‐related disorders (4821; 9.9%), mental health disorders (4314; 9.8%), asthma (4126; 8.5%), thyroid diseases (3576; 7.4%), endometrial disease (1927; 3.9%), cardiovascular disease (1525; 3.1%), and polycystic ovary syndrome (PCOS) (1464; 3.0%). While 22.5% of women had no medical conditions, 77.5% had one or more. Multimorbidity was associated with conditions including overweight, obesity, vitamin deficiency, thyroid disease, substance use, PCOS, GDM, and endometrial diseases. On cluster analysis, aged 35 years or older, overweight, vitamin deficiency, obesity, thyroid disease, asthma, uterine disease, other blood disorders, mental disorders, and PCOS were associated with GDM.

**Conclusions:**

More than three‐quarters of pregnant women in the Australian urban setting experienced one or more morbidities during pregnancy, which can be associated with adverse pregnancy outcomes. This project contributes to developing a learning health system infrastructure to deliver high‐value maternal health care while reducing costs.

## 1 INTRODUCTION

Pregnancy is a complex physiological process involving several critical stages, such as ovulation, fertilization, implantation, decidua formation, placentation, organogenesis, establishing the maternal‐fetal interface, fetal growth, and, ultimately, parturition.^1^ Completing each stage is vital for achieving a successful pregnancy. However, preexisting and pregnancy‐related medical conditions are prevalent and can interfere with these stages, often occurring as simultaneous comorbidity in pregnant women.^2^, ^3^, ^4^

Over time, the average maternal age for primigravidas increased from 28.4 years in 2011 to 29.7 years in 2021. For multigravidas, it rose from 31.3 years in 2011 to 32.2 years in 2021 in Australia, similar to other high‐income countries.^5^ There is also an alarming rise in average prepregnancy body mass index (BMI) globally.^6^ This upward trend of prepregnancy risk factors is accompanied by significant physiological and metabolic alterations^7^ and the development of chronic diseases, including type 2 diabetes mellitus (T2DM), hypertension, and dyslipidemia.^8^, ^9^ As a result, 56% of Australian women have at least one chronic condition.^10^

When a body that is already disturbed metabolically with chronic disease is exposed to pregnancy, which further alters body physiology,^11^ the preexisting metabolic condition might be exacerbated, or a woman may develop new metabolic disturbances like gestational diabetes mellitus (GDM) or preeclampsia.^12^, ^13^ GDM is a common, preventable cardiometabolic condition in pregnancy, affecting approximately 15% of women globally, with significant geographical variation and an increasing trend over time.^14^, ^15^ If such women remain undiagnosed, unmonitored, and untreated, it will often lead to maternal, fetal, and newborn complications, including cesarean births, low birth weight, preterm birth, stillbirths, and even death.^16^, ^17^ Therefore, it is essential to understand the distribution and interactions of common medical conditions (morbidities) and maternal characteristics in pregnancy. Identifying common contributing factors and adverse synergistic interactions between morbidities and maternal factors during pregnancy is essential to designing feasible and effective interventions. A key opportunity is to leverage electronic medical records (EMRs) to analyze the burden of these medical conditions, their interactions, and their impact on pregnancy outcomes.

Since maternal health is affected by different conditions emerging from a wide range of dimensions,^18^, ^19^, ^20^ health information from all parts of a woman's history could help inform the provision of optimal antenatal care.^21^, ^22^ Despite the existing technological advances, multidimensional, multi‐provider, and multidisciplinary cooperation and systems to provide comprehensive health information have not yet been fully implemented by existing healthcare systems in many countries, including Australia.^23^ A term first coined by the US Institute of Medicine (IOM) called the learning health system (LHS)^24^ provides a model to facilitate health systems to move toward providing the best care, personalized for each woman. LHS models envision developing a continuous learning health system by aligning science, analytics, informatics, incentives, and culture for continuous improvement and innovation, looking for the best practice by actively participating in all elements and gaining new knowledge from daily experience.^25^ In pregnancy care, such an LHS approach could conduct sophisticated analytics to extract relevant information from the electronic health records of a woman and provide this information in user‐friendly formats to be used by antenatal care providers to tailor the best high‐value care for the woman and her offspring.^26^

In line with advanced technologies, modern medicine increases human understanding of disease and produces innovative treatment strategies. However, due to the complex nature of some diseases and variations in patient characteristics, experimental studies are costly to conduct and slow to put into practice.^27^, ^28^ Therefore, the available health system is hungry for a system through which continuous and unbiased new knowledge flows and helps individual pregnancy care practice through daily experience.

The aim was to advance the LHS as a platform for automating information extraction from EMRs from an ethnically diverse cohort of pregnant women. The objective was to uncover the prevalence of and patterns between common morbidities during pregnancy and pregnancy‐related complications and identify clusters of comorbid conditions associated with GDM, the most common metabolic complication in pregnancy.^29^, ^30^ With rising maternal comorbidities and costs of maternity care in Australia,^31^ such information is timely, as investments and infrastructure for such LHS approaches will be necessary to inform and enable monitoring, prevention, and improved maternity care.

## 2 METHODS

### 2.1 Study population and data sources

The study utilized routinely gathered health data from pregnant women who received care at Monash Health maternity hospitals in Melbourne, Australia, between January 2016 and June 2021. Monash Health, one of Australia's most extensive public health networks, caters to over two million individuals throughout their lives. It encompasses eight hospitals and manages more than 12 000 births annually. As part of Australia's universal, freely accessible public healthcare system, Monash Health serves women from over 78 countries with a large immigrant population and 65% of mothers born overseas. Study findings are reported in line with the REporting of studies Conducted using the Observational Routinely‐collected Data (RECORD) statement.^32^

### 2.2 Data structure and terminologies

In this study, “morbidity” is defined as a preexisting medical condition, comorbidity as any coexisting medical condition, and multimorbidity as having two or more medical conditions. The term hypertension in this paper means preexisting medical conditions of hypertension. Pregnancy‐induced hypertension and preeclampsia are defined as pregnancy‐related complications. Semi‐structured data: documented in free text form in the dataset are factors such as maternal medical conditions, antenatal medications, substance use during pregnancy, obstetric complications, labor complications, indication for induction, birth defects, and immediate post‐labor complications. The data were recorded by clinicians, midwives, and data personnel working at the front line who are responsible for antenatal care. The standard operating procedure for this is that information deemed relevant to the current care is sought and entered into the medical record. This can be done by directly asking the woman questions, or it could be information volunteered by the woman herself. Semi‐structured is defined as data that falls between structured and unstructured data, has self‐descriptive properties, and is arranged with limited structural constraints, typically in the form of extended markup language.^33^ To enhance data accessibility, usability, and knowledge discovery, we applied basic Natural Language Processing (NLP) to convert unstructured text into a structured format. Structured data: The variables “overweight” (25.0 to <30 kg/m^2^) and “obesity” (≥ 30 kg/m^2^) were derived from BMI data, and “advanced age,” defined as an age of 35 years or older, was created using maternal age data.

### 2.3 Data cleaning and analysis

Analysis was performed in Python (version 3.10.5) using various packages. Since within the dataset, all columns record disease names only if the pregnant women have any condition and are left empty otherwise, we combined rule‐based extraction with normalization and fuzzy matching.

The pandas library was used for data manipulation and analysis. Text cleaning, sentence tokenization, and word replacement were facilitated by the *NLTK* package (version 3.7)^34^ and the *re* package (version 3.10.5)^35^ for handling regular expressions. Both *CountVectorizer* and *TfidfVectorizer* from the *sklearnfeature_extraction.text* module were utilized for converting text documents into matrices of token counts and *Term Frequency‐Inverse Document Frequency* (*TF‐IDF*) features, respectively. Topic modeling was performed using the Latent Dirichlet Allocation method from the *sklearn.decomposition* module, while the *sklearn.cluster* module's *KMeans* function enabled clustering analysis. The Spacy library, along with its Matcher module, was employed for advanced NLP tasks such as pattern matching. Data visualizations were created using the *matplotlib.pyplot* library and the *FreqDistVisualizer* from the *yellowbrick.text.freqdist* module.

The Natural Language Toolkit (NLTK) package was utilized for tasks such as tokenization, stemming, lemmatization, and parsing of texts. The “string” and “re” modules were employed for string formatting, searching, manipulation, and for dealing with regular expressions. The text underwent a cleaning process to remove punctuations, numbers, and whitespaces. Additionally, all words were converted to lowercase for consistency. Word tokenization was then performed to split the text into separate words or tokens, enabling a better understanding of the context. To enhance the quality of analysis, common stop words (e.g., “*a,” “an,” “and,” “are,” “as,” “at,” “be,” “by,” “for,” “from,” “has,” “he,” “in,” “is,” “it,” “its,” “of,” “on,” “that,” “the,” “to,” “was,” “were,” “will,” “with*,” etc.) that have little contribution to the overall meaning of the text and do not contribute significant meaning to the analysis were eliminated from the text data. To gain insights into the distribution and importance of words, the frequencies of the most common words were calculated. This analysis provided an overall understanding of the prominence and relevance of words in the text data. Finally, the text data were visualized using WordCloud, a technique that presents a quick overview of key terms and themes. The WordCloud representation allows for visual identification of the most prominent words, enabling viewers to grasp the main focus or subject of the text rapidly. The size of each word in the WordCloud is related to the frequency of the word (larger font indicates greater frequency), and it provided an important visual figure that enabled us to get an overarching insight into relevant themes and to identify important keywords before further analysis.

Keywords for each common medical condition were extracted by examining the top 1000 most frequently mentioned medical conditions. As an example, for mental disorders, the identified keywords included “anxiety,” “depression,” “panic,” “psychological,” “stress,” “bpad,” “bipolar,” “personality,” “borderline,” “schizophrenia,” “intellectually,” “bpd,” “fatigue,” “ptsd,” “psychosis,” “bulimia,” “psych,” “substance,” “suicidal,” “borderline,” and “schizoaffective.” Here, “bpad” refers to bipolar disorder; “bpd” to borderline personality disorder; and “ptsd” to post‐traumatic stress disorder. These keywords were used to determine whether there was a diagnosis of a mental disorder in a given patient record. The “Spacy” matcher Python module was utilized for this purpose. Keywords used to extract each disease are indicated in the Data S1.

Due to the nature of the dataset, which does not include negative or uncertain contexts, we did not find it necessary to use more advanced NLP techniques to handle negative and uncertain contexts. We rather employed rule‐based extraction using MedSpaCy with TargetRule for disease extraction. This method is straightforward and effective for predefined lists of terms, but it may miss variations not covered by the rules. To avoid missing any conditions, we printed the top 1500 words available in the columns and extracted all relevant terms to capture variations. We used normalization to convert text to lowercase, ensuring consistency and reducing variability due to capitalization. We then applied fuzzy matching using the FuzzyWuzzy library to handle minor typos and variations. This approach is useful for matching terms that are similar but not exact, which is important for dealing with real‐world data where spelling errors are common.

The frequencies of the most common words were calculated to gain insights into the distribution and importance of words. Common medical conditions were extracted from the text based on word frequency.

After extracting common medical conditions, their associations with GDM and common obstetric, labor, and fetal complications were calculated. The chi‐square statistic was calculated to assess the degree of association between morbidities and each pregnancy complication. Morbidity comparisons were also performed between (a) pregnant women who had GDM and (b) pregnant women who had no GDM. A *p*‐value of <0.05 was considered statistically significant. To understand the strength of the association, we used the Phi coefficient, which is derived from the chi‐square statistic.

### 2.4 Cluster analysis

Cluster analysis was performed to group a set of factors so that those in the same group (or cluster) are more similar to each other than those in different groups. The K‐means clustering algorithm was applied, and the elbow method determined the number of clusters. As the most common cardiometabolic complication in pregnancy and the context of a research program focused on GDM risk identification, prevention, and management, the risks of GDM across various clusters were also assessed after creating isolated columns representing clusters.

## 3 RESULTS

**FIGURE 1:**
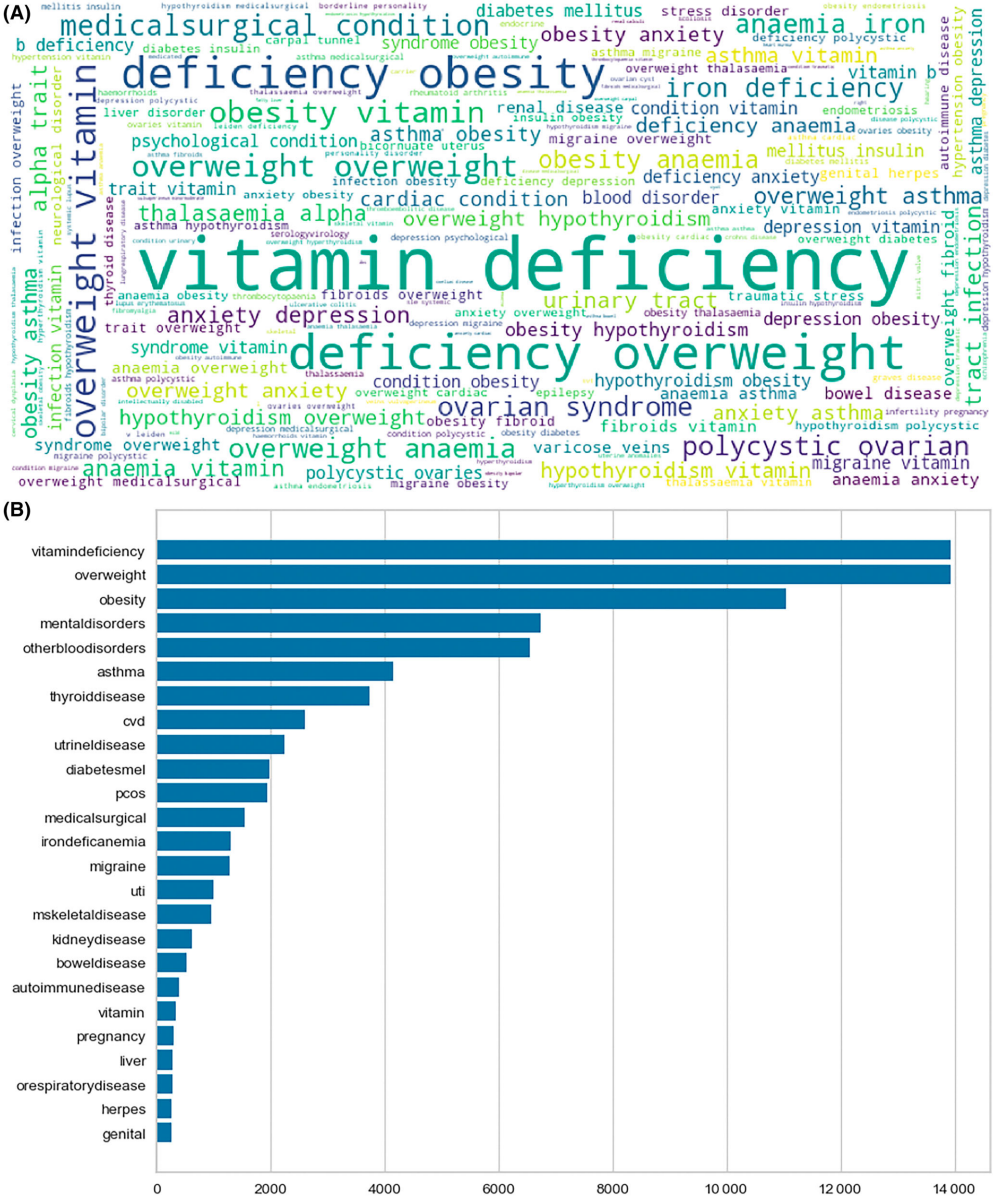
Common pregnancy comorbidities. (A) Top panel: WordCloud of common pregnancy comorbidities. (B) Bottom panel: Top 25 commonly occurring medical condition tokens.

This analysis incorporated a total of 48 502 pregnancies. More than half of the participants had a BMI exceeding 25 kg/m^2^, and 22.5% were over the age of 35 years. Common medical conditions were vitamin deficiency (*n* = 14 019; 28.9%), overweight (*n* = 13 918; 28.7%), obesity (*n* = 11 026; 22.7%), anemia and other blood‐related disorders (*n* = 4821; 9.9%), mental health disorders (*n* = 4314; 9.8%), asthma (*n* = 4126; 8.5%), thyroid diseases (*n* = 3576; 7.4%), endometrial disease (*n* = 1927; 3.9%), cardiovascular disease (CVD) (*n* = 1525; 3.1%), PCOS (*n* = 1464; 3.0%), iron deficiency anemia (*n* = 1308; 2.7%), migraine (*n* = 1274; 2.6%), urinary tract infections (*n* = 984; 2.0%), diabetes mellitus (*n* = 743; 1.53%), musculoskeletal disease (*n* = 666; 1.4%), hypertension (*n* = 581; 1.2%), and kidney diseases (*n* = 392; 0.8%) (Figure 1).

Common complications included GDM (*n* = 10 343; 21.3%), induced birth (*n* = 14 623; 30.1%), cesarean section (n = 14 027; 28.9%), admission of newborn to special care nursery or antenatal care unit (*n* = 8848; 18.1%), macrosomia (*n* = 4308; 8.9%), preterm delivery (*n* = 4066; 8.4%), preeclampsia (*n* = 1698; 3.5%), shoulder dystocia (*n* = 1069; 2.2%), and birth defect (*n* = 2464; 5.1%).

### 3.1 Frequency of single morbidity and multimorbidity

The number of conditions experienced during pregnancy ranged from none to nine. A relatively low proportion of women (*n* = 10 890; 22.5%) had no captured conditions, while (*n* = 18 143; 37.4%) had one medical condition and (*n* = 12 467; 25.7%) had contended with two conditions. The percentage continued to decline as the number of conditions increased, with (*n* = 5131; 10.6%) having three conditions, (*n* = 1385; 2.9%) having four, and (*n* = 1; <0.1%) having nine conditions (Figure S1).

In the total cohort, (*n* = 37 612; 77.5%) had ≥1 comorbidity during pregnancy, of which (*n* = 18 143; 37.4%) had a single medical condition while (*n* = 19 469; 40.1%) had multimorbidity. Of 10 913 pregnant women aged 35 years and above, this increased to (*n* = 8731; 18.0%) having ≥1 comorbidity, with (*n* = 3932; 8.1%) experiencing a single morbidity, while (*n* = 4799; 9.9%) presented with multimorbidity. All 24 943 pregnant women with a BMI of ≥25 kg/m^2^ had ≥1 comorbidity, with (*n* = 9525; 19.6%) having a single morbidity and (*n* = 15 418; 31.8%) with multimorbidity (Table 1).

**TABLE 1:** The distribution of comorbidities across baseline characteristics of participants and selected obstetric complications.

Characteristics	Total births (N = 48 502)	No morbidity (*N* = 10 890)	Single morbidity (*N* = 18 143)	Multimorbidity^a^ (*N* = 19 469)
Age (years)				
≤24	6176 (12.7%)	1347 (12.4%)	2348 (12.9%)	2481 (12.7%)
25–29	13 806 (28.5%)	3239 (29.7%)	5243 (28.9%)	5324 (27.3%)
30–34	17 607 (36.3%)	4122 (37.9%)	6620 (36.5%)	6865 (35.3%)
35–39	8830 (18.2%)	1823 (16.7%)	3191 (17.6%)	3816 (19.6%)
≥40	2083 (4.3%)	359 (3.3%)	741 (4.1%)	983 (5.0%)
BMI (kg/m^2^)				
≤19.9	4169 (8.6%)	1976 (18.1%)	1503 (8.3%)	690 (3.5%)
20–24.9	19 144 (39.5%)	8798 (80.8%)	7042 (38.8%)	3304 (17.0%)
25–26.9	6632 (13.7%)	0 (0.0%)	2785 (15.4%)	3847 (19.8%)
27–29.9	7286 (15.0%)	0 (0.0%)	2969 (16.4%)	4317 (22.2%)
30–34.9	6276 (12.9%)	0 (0.0%)	2301 (12.7%)	3975 (20.4%)
35+	4749 (9.8%)	0 (0.0%)	1470 (8.1%)	3279 (16.8%)
Ethnicity				
Caucasian	1852 (3.8%)	166 (1.5%)	713 (3.9%)	973 (5.0%)
Oceania (not White‐Australian or White‐New Zealander)	21 256 (43.8%)	4057 (37.3%)	7683 (42.3%)	9516 (48.9%)
Middle‐Eastern, North African, or Sub‐Saharan African	2967 (6.1%)	549 (5.0%)	1159 (6.4%)	1259 (6.5%)
Southern and Central Asian	14 549 (30.0%)	3108 (28.5%)	5588 (30.8%)	5853 (30.1%)
South‐East and North‐East Asian	7778 (16.0%)	2989 (27.4%)	2970 (16.4%)	1819 (9.3%)
Other	100 (0.2%)	21 (0.2%)	30 (0.2%)	49 (0.3%)
Parity				
0	19 357 (39.9%)	4583 (42.1%)	7166 (39.5%)	7608 (39.1%)
1	17 618 (36.3%)	4231 (38.9%)	6626 (36.5%)	6761 (34.7%)
≥2	11 527 (23.8%)	2076 (19.1%)	4351 (24.0%)	5100 (26.2%)
Substance use	6033 (12.4%)	878 (8.1%)	2091 (11.5%)	3064 (15.7%)
Preeclampsia	1698 (3.5%)	192 (1.8%)	586 (3.2%)	920 (4.7%)
Pregnancy‐induced Hypertension	1113 (2.3%)	102 (0.9%)	409 (2.3%)	602 (3.1%)
Gestational diabetes mellitus	10 343 (21.3%)	1608 (14.8%)	3768 (20.8%)	4967 (25.5%)
Preterm birth	4066 (8.4%)	783 (7.2%)	1413 (7.8%)	1870 (9.6%)
Induction of labor	14 623 (30.1%)	2962 (27.2%)	5257 (29.0%)	6404 (32.9%)
Cesarean delivery	14 027 (28.9%)	2415 (22.2%)	5064 (27.9%)	6548 (33.6%)
Birth defect	2464 (5.1%)	537 (4.9%)	918 (5.1%)	1009 (5.2%)
Admission to a special nursery or neonatal intensive care unit	8848 (18.1%)	1604 (14.7%)	3117 (17.2%)	4127 (21.2%)
Shoulder dystocia	1069 (2.2%)	210 (1.9%)	415 (2.3%)	444 (2.3%)
Macrosomia	4308 (8.9%)	701 (6.4%)	1721 (9.5%)	1886 (9.7%)

^a^ Having ≥2 comorbidities.

### 3.2 Association of morbidities with complications

**FIGURE 2:**
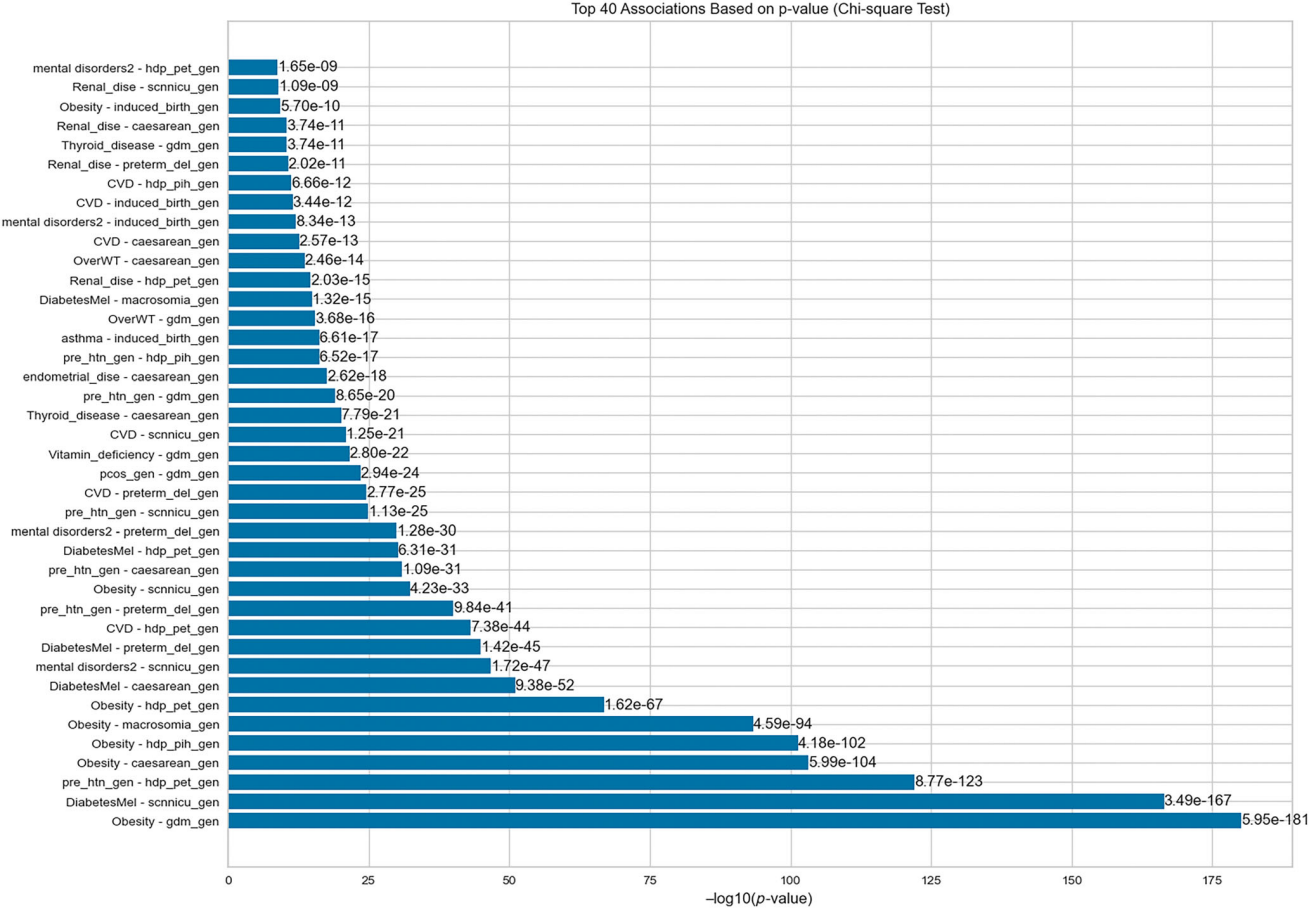
Top 40 associations based on *p*‐value (Chi‐squared test). The level of statistical significance decreases from bottom to top. CVD, cardiovascular disease; D.M., diabetes mellitus; GDM, gestational diabetes mellitus; PCOS, polycystic ovary syndrome; PIH, pregnancy‐induced hypertension; SCN_NICU, admission of newborn to special care nursery or antenatal care unit.

The top 40 significant correlations are presented in Figures 2 and S2. It shows critical associations such as obesity with GDM, macrosomia, hypertensive disorders of pregnancy, preeclampsia, and cesarean section; preexisting diabetes with neonatal special care admission and type of delivery; and preexisting maternal hypertension with preeclampsia, among others.

**FIGURE 3:**
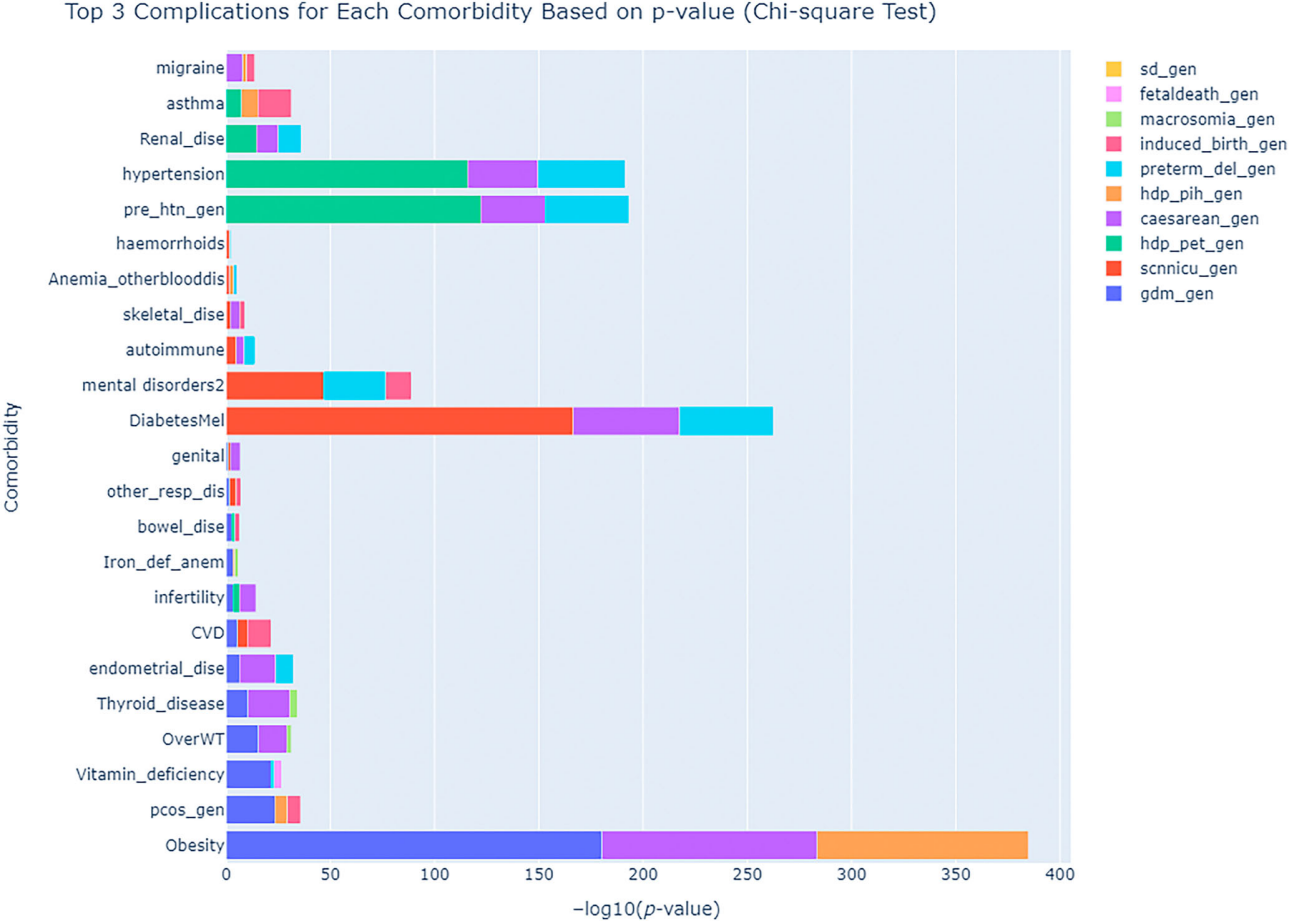
The top three complications associated with each comorbidity. CVD, cardiovascular disease; D.M., diabetes mellitus; GDM, gestational diabetes mellitus; PCOS, polycystic ovary syndrome; PIH, pregnancy‐induced hypertension; SCN_NICU, admission of newborn to special care nursery or antenatal care unit.

The top three complications associated with each morbidity are presented in Figure 3. Overweight, obesity, vitamin deficiency, thyroid disease, substance use, PCOS, and endometrial diseases are among the morbidities most associated with GDM (Table 2).

**TABLE 2:** Medical conditions and their association with gestational diabetes mellitus.

Medical conditions	Gestational diabetes		Chi‐squared	*p*‐value
	No	Yes		
Overweight^a^				
No	27 542 (56.8)	7042 (14.5)	66.4	< 0.001
Yes	10 617 (21.9)	3301 (6.8)		
Obesity^a^				
No	30 570 (63.0)	6907 (14.2)	822.8	< 0.001
Yes	7589 (15.6)	3436 (7.1)		
Common mental disorders				
No	34 716 (71.6)	9472 (19.5)	3.6	0.056
Yes	3443 (7.1)	871 (1.8)		
Polycystic ovary syndrome				
No	37 164 (76.6)	9874 (20.4)	103.3	< 0.001
Yes	995 (2.1)	469 (1.0)		
Cardiovascular disease				
No	36 991 (76.6)	9986 (20.6)	3.9	0.046
Yes	1168 (2.4)	357 (0.7)		
Substance use				
No	33 079 (68.2)	9390 (19.4)	125.1	<0.001
Yes	5080 (10.5)	953 (2.0)		
Thyroid disease				
No	35 502 (73.2)	9424 (19.4)	43.7	<0.001
Yes	2657 (5.5)	919 (1.9)		
Urinary tract infections				
No	37 386 (77.1)	10 132 (20.9)	0.0	0.958
Yes	773 (1.6)	211 (0.4)		
Hypertension				
No	37 792 (77.1)	10 129 (20.9)	83.4	< 0.001
Yes	367 (0.8)	214 (0.4)		
Vitamin deficiency				
No	27 527 (56.8)	6956 (14.3)	94.2	<0.001
Yes	10 632 (21.9)	3387 (7.0)		
Asthma				
No	34 871 (71.9)	9505 (19.6)	2.7	0.100
Yes	3288 (6.8)	838 (1.7)		
Other respiratory disease				
No	37 978 (78.3)	10 273 (21.2)	6.1	0.013
Yes	181 (0.4)	70 (0.1)		
Anemia and other blood disorders				
No	34 399 (70.9)	9282 (19.1)	1.4	0.2
Yes	3760 (7.8)	1061 (2.2)		
Iron deficiency anemia				
No	37 079 (76.4)	10 115 (20.9)	0.6	0.440
Yes	1080 (2.2)	228 (0.5)		
Endometrial disease				
No	36 732 (75.7)	9843 (20.3)	25.3	<0.001
Yes	1427 (2.9)	500 (1.0)		
Migraine				
No	37 182 (76.7)	10 046 (20.3)	1.0	0.314
Yes	977 (2.0)	297 (0.6)		
Kidney disease				
No	37 829 (78.0)	10 281 (21.2)	6.8	0.009
Yes	330 (0.7)	62 (0.1)		
Bowel disease				
No	37 903 (78.1)	10 301 (21.2)	8.9	0.0028
Yes	256 (0.5)	42 (0.1)		
Genital herpes				
No	37 899 (78.1)	10 289 (21.2)	2.9	0.085
Yes	260 (0.5)	54 (0.1)		
Hemorrhoids				
No	37 963 (78.3)	10 282 (21.2)	0.8	0.384
Yes	196 (0.4)	61 (0.1)		
Autoimmune disease				
No	37 897 (78.1)	10 270 (21.2)	0.0	0.887
Yes	262 (0.5)	73 (0.2)		
Musculoskeletal disease				
No	37 628 (77.6)	10 208 (21.0)	0.4	0.534
Yes	531 (1.1)	135 (0.3)		
Infertility				
No	38 077 (78.5)	10 300 (21.2)	12.0	<0.001
Yes	82 (0.2)	43 (0.1)		
Multimorbidity				
No	23 612 (48.7)	5356 (11.0)	344.3	<0.001
Yes	14 547 (30.0)	4987 (10.0)		
Advanced age (≥35 years)				
No	30 476 (62.8)	7113 (14.7)	573.8	<0.001
Yes	7683 (15.8)	3230 (6.7)		

^a^ Overweight and obesity information were determined from prepregnancy BMI data and were mutually exclusive.

### 3.3 Cluster analysis

**FIGURE 4:**
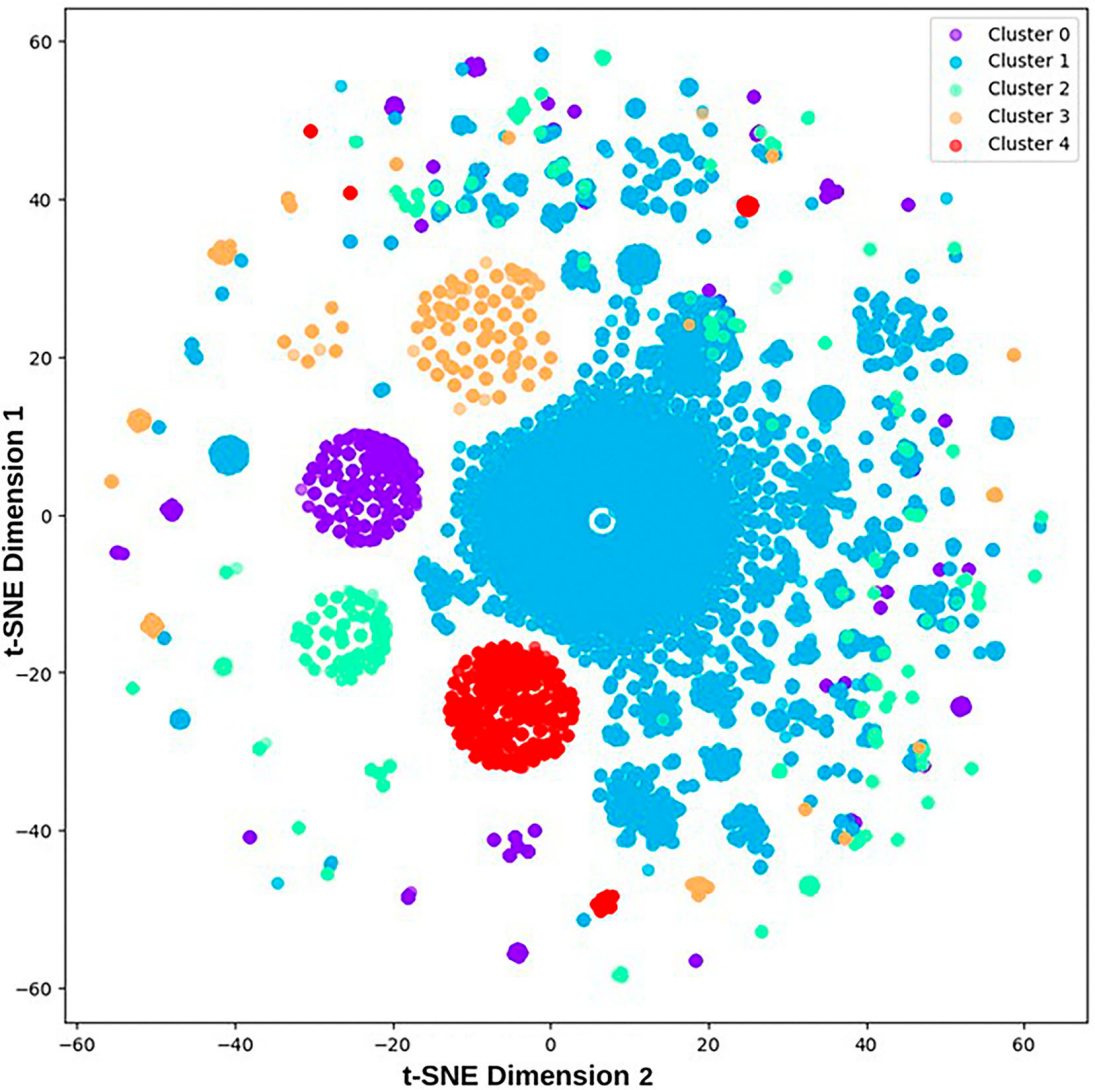
The cluster analysis results scatter plot shows those who share similar comorbidities during their pregnancies (each point representing a pregnant woman). Cluster 0: obesity, vitamin deficiency, asthma, other blood disorders, advanced age, mental disorders, thyroid disease, PCOS, migraine, urinary tract infection; Cluster 1: mental disorders, other blood disorders, asthma, thyroid disease, overweight, vitamin deficiency, uterine disease, PCOS, iron deficiency anemia, obesity; Cluster 2: advanced age, overweight, vitamin deficiency, obesity, thyroid disease, asthma uterine disease, other blood disorders, mental disorders, PCOS; Cluster 3: overweight, vitamin deficiency, thyroid disease, other blood disorders, asthma, mental disorders, PCOS, uterine disease, migraine, cardiovascular disease; Cluster 4: vitamin deficiency, other blood disorders, thyroid disease, asthma, mental disorders.

The text clustering analysis resulted in five distinct clusters based on the medical conditions of pregnant women. Figure 4 presents the cluster analysis visually.

In assessing the risk of GDM across various clusters, it was found that 29.4% of women in Cluster 0 developed GDM, 17.5% in Cluster 1, 29.5% in Cluster 2, 21.5% in Cluster 3, and 14.6% in Cluster 4. There was no significant difference in the incidence of GDM between clusters 2 and 0 (*p* = 0.86). However, statistically significant differences between the remaining cluster combinations indicated varying GDM risks among the clusters.

## 4 DISCUSSION

We extracted preexisting and pregnancy‐related medical conditions in pregnant women, and much of this information was found in semi‐structured format in EMRs. Overall, the extracted information showed that the burden of morbidity was high. The most common morbidities during pregnancy included vitamin deficiency, overweight, obesity, anemia and other blood‐related disorders, mental health disorders, asthma, thyroid diseases, endometrial disease, CVD, PCOS, and iron deficiency anemia. Associations such as obesity with GDM, macrosomia, hypertensive disorders of pregnancy, preeclampsia, and cesarean section; preexisting diabetes with neonatal special care admission and type of delivery; and preexisting maternal hypertension with preeclampsia, among others, were observed.

To advance our large public antenatal services in Australia toward an LHS infrastructure that can automate information extraction from EMRs, this work was codeveloped with our multidisciplinary clinician coauthors, including an obstetrician (D.L.R.), a midwife (M.B.K.), two endocrinologists (H.T., R.G.), and a dietitian (L.M.), who provide expert care to pregnant women. This study outlines the service and patient outputs that frontline clinicians require in a timely manner from an LHS infrastructure. Ideally, the work conducted in this project would be automated within an LHS to provide regular updates on these outputs, allowing clinicians and administrators to monitor outcomes and implement both systemic (organization‐wide) and individual interventions as needed. Clinicians have also indicated that disaggregating data by each treating clinician or team would be valuable, provided it occurs in an environment that supports professional development and is not used for punitive purposes.^36^ Ultimately, embedded analytics within an LHS would utilize this data to enable predictive risk stratification of high‐risk patients and real‐time clinical decision support for tailored patient interventions. The curated data were used as inputs for developing and temporally validating our risk prediction models,^29^, ^37^, ^38^ which are intended to enhance decision‐making in personalized maternity care within our LHS.

The number of medical conditions during pregnancy varied from none to as many as nine. Our results indicated that a significant proportion, approximately 77.5%, of pregnant women experience at least one morbidity during pregnancy. Of these, 37.4% had a single medical condition, while 40.1% had multimorbidity. This finding is consistent with an emerging body of evidence that recognizes the high prevalence of morbidities among pregnant women.^39^ In contrast, other studies have shown the prevalence of multimorbidity ranging from 0.83% to 46.2%, which is lower than our findings.^40^, ^41^, ^42^, ^43^ This might be due to differences in the characteristics of the populations studied, inclusion and exclusion criteria, and the spectrum of medical conditions considered in defining morbidity.

This high level of morbidity, combined with advanced maternal age, contributes to the rapidly rising rates of pregnancy complications, including induction of labor and cesarean delivery.^44^ The health burden and cost of these conditions and the related pregnancy impact are substantial, with recent evidence showing a 60% increase in Australian public maternity costs over the last 5 years alone.^44^ This is unsustainable, and understanding these morbidities will assist with prevention. For example, lifestyle interventions, which are cost‐effective and recommended for implementation for prevention, are proven to reduce many of these complications in pregnancy.^15^, ^31^, ^45^ Implementation remains limited, with a persistent focus on costly treatments. Appreciation of these data also recognizes the need to significantly optimize healthcare services during pregnancy to protect Australia's mothers and babies.

The variability in the number of morbidities during pregnancy was striking, with 22.5% of women not experiencing any medical conditions. While this suggests many pregnancies may be categorized as low‐risk, 37.4% of women had one medical condition, and 25.7% had two, necessitating medical attention.^46^ About 14% had three or more medical conditions, and this number is significantly higher when looking at subgroups with high BMI and increased age. This suggests that to achieve the most significant impact, these groups are targeted with more comprehensive antenatal care capable of managing multiple concurrent conditions.^47^ It has been reported that advanced maternal age is more likely to be associated with a BMI of 25 or higher, as well as with other comorbid conditions.^17^, ^48^

The NLP methods applied to the EMRs can enhance the learning health system loop by transforming semi‐structured clinical data into structured, actionable data, which enables the extraction of valuable clinical insights that can be used for decision support, research, and improving patient care.^49^ It enables the unlocking of previously hidden clinical information, increases data heterogeneity, and aids in predicting and preventing diseases.^50^ Integrating NLP with machine learning models, such as logistic regression and BERT, has shown high efficiency in diagnosing disease groups and can be used for preliminary health assessments.^51^ Deep learning and convolutional neural networks further enhance the ability to detect relationships between diseases, symptoms, and treatments, thereby facilitating better clinical decision‐making.^52^ By developing annotation‐free NLP pipelines, health systems can automatically derive precise clinical codes from pathology reports, thus enabling a self‐learning system that continuously improves from historical and emerging data.^53^

### 4.1 Implications of the study and the learning health system

Our study offers valuable insights into the prevalence and associations of morbidities and maternal characteristics with obstetric complications, thereby enriching the field of obstetric research. Despite the increasing prevalence of morbidity risk factors like advanced maternal age and obesity, healthcare services are generally designed around managing individual chronic conditions.^54^ Since the collective impact of multimorbidity differs significantly from that of a single condition, effective preconception management and maintaining a coordinated and focused healthcare approach throughout the antenatal period are required to prevent unfavorable outcomes. Evidence from non‐pregnancy contexts suggests collaborative, multidisciplinary approaches effectively manage multimorbidity.^54^ These include guidelines and strategies for dealing with common clusters of conditions, necessitating a deep understanding of their co‐occurrence and potential issues like drug–drug interactions.^55^, ^56^ The development and implementation of such strategies are vital, given the increasing prevalence of multimorbidity risk factors. The ability to gather valuable data from semi‐structured EMRs using NLP could support shared decision‐making and save resources as unnecessary time‐intensive tasks and repeated tests are avoided. Critical multimorbidity data are made readily available.^26^, ^57^

The structured data from this study enabled us to analyze frequency, trends, and associations between risk factors and pregnancy complications and explore new insights; beyond associations, we also conducted clustering analyses, which are vital exploratory tools that can uncover new patterns and insights. The findings from frequency associations, trend analyses, and clustering results helped to advance our precision medicine platform for personalized pregnancy care. This study enabled us to identify potential morbidities within the semi‐structured EMRs that previously were not being considered in our existing work program, which focused on risk prediction for GDM and improving pregnancy and birth outcomes, therefore improving the number and accuracy of potential predictors. Finally, the study allowed us to consider interacting mechanisms and gateways that may link clustered conditions to adverse outcomes in pregnancy. Finally, this study is part of a more extensive program that builds an LHS to deliver high‐value maternal health care and personalized pregnancy care.^26^, ^57^

### 4.2 Strengths and limitations

This study involved an extensive evaluation of morbidities in a large sample size of pregnant women by leveraging EMRs and NLP text mining. However, the study has some limitations. Firstly, measurement bias may arise from inaccuracies or errors during data collection, especially as the data were routinely collected for clinical purposes. Furthermore, the statistical measures reflect the relationship between variables and do not necessarily represent causal associations. Hence, these findings, while helpful in advancing our understanding of morbidities in pregnancy, warrant cautious interpretation.

## 5 CONCLUSIONS

Three‐quarters of pregnant women experienced at least one medical condition, of which 40% had two or more. In the context of rapidly rising pregnancy interventions, costs, and adverse outcomes, this study emphasizes the importance of considering the entire health profile of patients when predicting and managing risk and the need for public health measures and comprehensive prenatal and pregnancy care. GDM was common during pregnancy, affecting more than 1‐in‐5, and our results highlight the strong associations between several comorbidities and the incidence of GDM, which will guide prevention efforts. This work was codeveloped with our multidisciplinary clinicians, and is an important step in the pipeline that will ultimately deliver automated information extraction from EMRs within an LHS to drive better care and outcomes.

## AUTHOR CONTRIBUTIONS

Y.B., J.E., H.T., L.M., and A.M. were involved in acquiring data, conceptualization, methodology, software, data curation, formal analysis, and writing—the original draft. All the remaining authors were involved in interpreting the data and critically reviewing or revising the manuscript for important intellectual content. All authors approved the final version for publication.

## FUNDING INFORMATION

There was no funding for this project. Y.B. is supported by a Monash Graduate Scholarship (MGS) and Monash International Tuition Scholarship (MITS). A.M. and H.T. are funded by National Health and Medical Research Council (NHMRC) Fellowships.

## CONFLICT OF INTEREST STATEMENT

The authors declare no conflict of interest.

## ETHICS STATEMENT

Ethics approval was obtained from Monash Health with application number of RES‐21‐0000‐183 L. The research has been conducted in adherence with the Code of Ethics of the World Medical Association, also known as the Declaration of Helsinki.

## Supporting information


Data S1.

## Data Availability

All data and material access requests can be forwarded to the corresponding author's email address.
