# Supplementary material for: Analyzing electronic medical records to extract prepregnancy morbidities and pregnancy complications: Toward a learning health system
Source: Learn Health Syst. 2024 Nov 26;9(2):e10473. doi: 10.1002/lrh2.10473 (PMC12000771; doi:10.1002/lrh2.10473)
Supplement: Supplementary file 1 — Data S1. [file LRH2-9-e10473-s001.docx]

**Supplementary file**

**Supplementary Results**


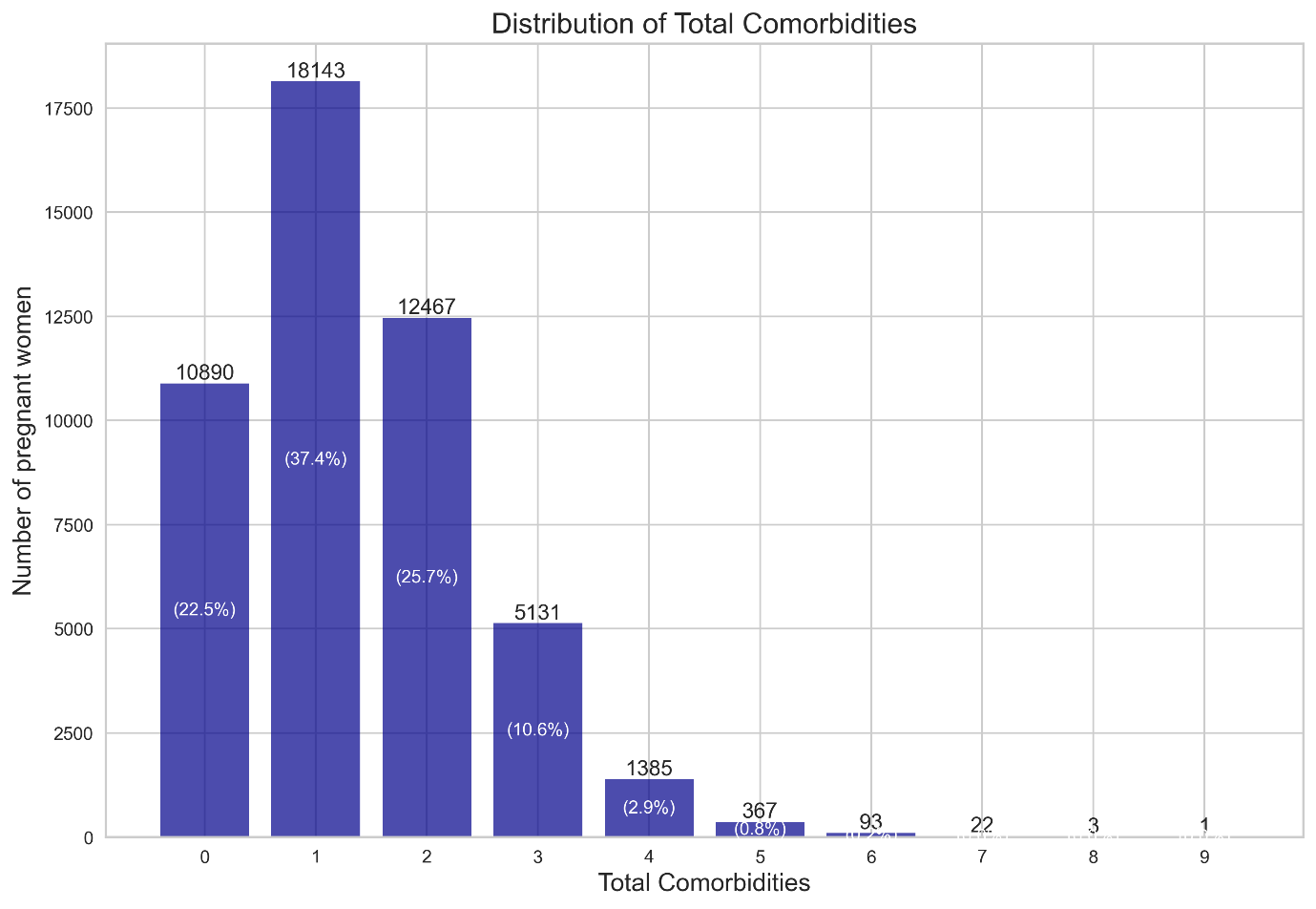


**Figure S1.** Bar graph showing the frequency of comorbidities during pregnancy.


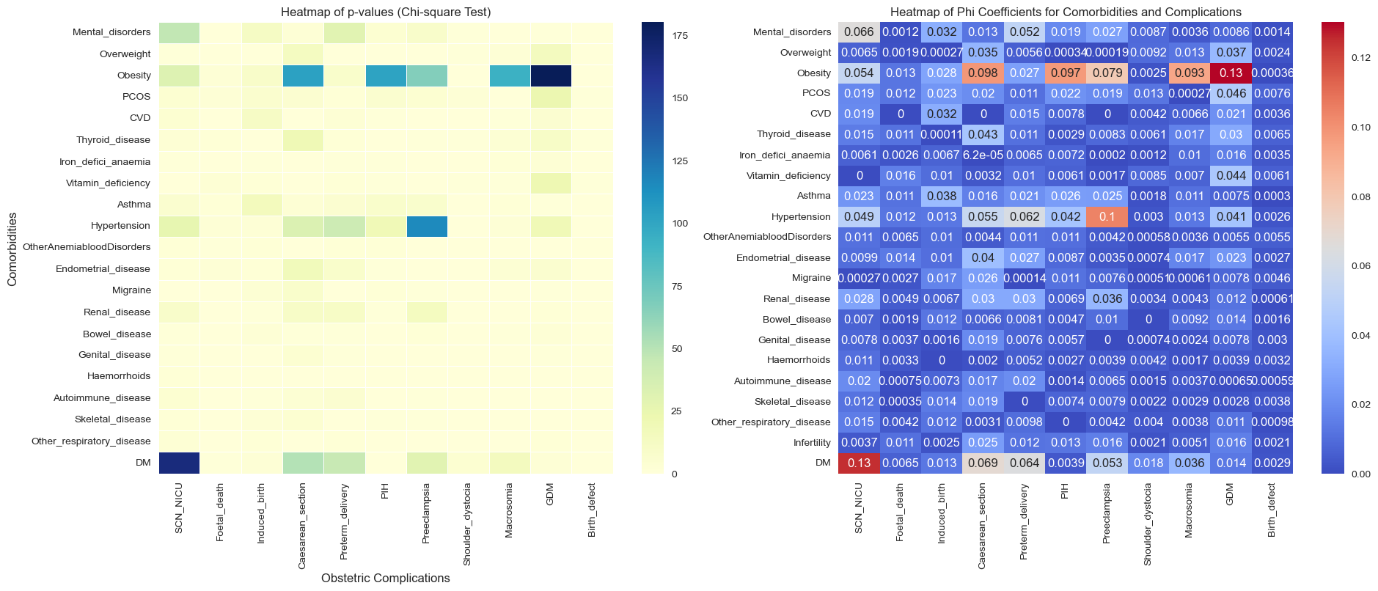


**Figure S2**. Heatmaps of Chi-squared analysis results and Phi coefficients.

**Abbreviations**: *PCOS: polycystic ovary syndrome, CVD: cardiovascular disease, DM: diabetes mellitus, SCN_NICU: admission of newborn to special care nursery or antenatal care unit, PIH: pregnancy induced hypertension, GDM: gestational diabetes mellitus*

**Footnote: Heatmap of Chi squared** *(The color of each cell represents the strength of the association between that pair of comorbidity and complication, as measured by the p-value. The color scale is provided in the legend of the heatmap. Darker colors (towards top end of the color spectrum) indicate a stronger association, while lighter colors (towards the bottom end of the spectrum) indicate a weaker association.)* **Heatmap based on Phi coefficients** (*The color of each cell represents the strength of the association between that pair of comorbidity and complication, as measured by the Phi coefficient. The color scale is provided in the legend of the heatmap. Darker colors (towards one end (top) of the color spectrum) indicate a stronger association, while lighter colors (towards the other end (bottom) of the spectrum) indicate a weaker association.)*

**Key terms used for common medical condition extraction**

**Polycystic ovary syndrome** = 'polycystic.*ovary.*disease', 'polycystic.*ovary.*syndrome', 'polycystic.*ovarian.*syndrome','pcos'

**Cardiovascular disease** = ['cardiac', 'hypertension', 'veins', 'varicose', 'heart', 'valve', 'svt', 'tachycardia', 'thromboembolitic', 'aortic', 'dvt', 'atrial', 'embolism', 'ventricular', 'cardiomyopathy', 'vsd', 'vein', 'arrhythmia', 'thrombosis', 'pacemaker', 'rheumatic', 'aorta', 'thrombocytosis', 'systolic', 'tricuspid', 'endocarditis', 'murmer', 'murmur','carotid','vascular','heartbeat','dextrocardia','arterial','pericardial','bicuspid','murmur']

**Musculoskeletal system disease** = ['arthritis', 'skeletal', 'osteoarthritis','scholiosis','osteogenesis','osteoporosis','osteo','carpal','arteritis','bone','joint','knee','back','scoliosis','spondylolisthesis','spondylosis', 'spondylitis', 'myasthenia','muscular']

**Other Respiratory disease** = ['tuberculosis', 'pulmonary', 'lungrespiratory','bronchitis','influenza','lung','apnea']

**Diabetes mellitus** = ['diabetes','insulin', 'mellitus', 'mellitis', 'diabetic']

**Mental disorders** = ['anxiety', 'depression', 'panic', 'psychological', 'stress', 'bpad', 'bipolar', 'personality', 'borderline', 'schizophrenia', 'intellectually', 'bpd', 'fatigue', 'ptsd', 'psychosis', 'bulimia', 'psych', 'substance', 'suicidal', 'boarderline', 'schizoaffective']

**Thyroid disease** = ['hypothyroidism', 'hyperthyroidism', 'thyroid', 'thyroidectomy', 'thyroiditis', 'palpitations', 'thyroxine', 'thyroditis', 'thyrotoxicosis', 'hemithyroidectomy', 'parathyroid', 'hyperparathyroidism', 'hyperparathyroidism']

**Anemia and other blood disorders** = ['anaemia', 'thalasaemia', 'thalassaemia', 'thrombocytopaenia', 'haemoglobin', 'prothrombin', 'haemorrhage', 'bleeding', 'blood', 'thalassemia']

**Iron deficiency anemia** : 'iron.*deficiency|haemochromotosis'

**Kidney disease** = ['renal', 'kidney', 'pyelonephritis','hydronephrosis','nephropathy','nephritis','nephrotic','hydronephritis','nephrectomy','nephrostomy','glomerulonephritis','nephrostomy']

**Endometrial disease** = ['endometriosis', 'endometrial', 'endometrioma','fibroids','uterus','uterine','bicornuate']

**Vitamin_deficiency**='vitamin.*deficiency'

**Asthma**= 'asthma'

**Migraine**= 'migraine'

**Genital herpes** = 'genital'

**Haemorrhoids**= 'haemorrhoids'

**Infertility**= ‘infertility’

**Urinary tract infections**='urinary.*tract.*infection',

**Autoimmune disease**='autoimmune.*disease|sle|lupus'

**Bowel disease** = 'bowel','crohns','ulcerative','colitis'
